# Entropy, Ecology and Evolution: Toward a Unified Philosophy of Biology

**DOI:** 10.3390/e25030405

**Published:** 2023-02-23

**Authors:** Samuel A. Cushman

**Affiliations:** Department of Biology, University of Oxford, Oxford OX1 2JD, UK; sam.cushman@gmail.com

**Keywords:** entropy, thermodynamics, ecosystems, ecology, dissipative structures, evolution

## Abstract

Darwin proposed that the capacity of organisms to produce more offspring that can be supported by the environment would lead to a struggle for existence, and individuals that are most fit for survival and reproduction would be selected through natural selection. Ecology is the science that studies the interaction between organisms and their environment within the context of Darwinian evolution, and an ecosystem is defined as a community of living organisms in conjunction with the nonliving components of their environment, interacting as a system. One topic that has been very much understudied and largely ignored in evolutionary biology is the overarching context of thermodynamics in controlling all biological processes and the evolution of life. Most fundamentally, organisms are self-replicating dissipative structures. Evolution is the process whereby variation in the structure of organisms have differential fitness in terms of their effectiveness at building and maintaining their structure, efficiently consuming free energy, and effectively reproducing and passing on those heritable variations, leading to change in the frequency of genetic variation and associated change in the characteristics in the population. The central process is dissipation of free energy according to the second law of thermodynamics, and evolution therefore is better conceptualized as the emergence of self-replicating dissipative structures that through natural selection become increasingly more efficient at degrading free energy. Ecosystems are linked series of dissipative structures with heat engine dynamics driven by random dissipation of energy and increasing entropy. The structure and composition of ecosystems across scales are emergent dissipative structures driven by the flow of energy and the increase in entropy. Communities and ecosystems are emergent properties of a system that has evolved to most efficiently dissipate energy and increase entropy. By focusing on the fundamental entity (energy), and the fundamental process (dissipation and disordering of energy and increasing of entropy), we are able to have a much clearer and powerful understanding of what life is, from the level of biochemistry, to evolution, to the nature of the organism itself, and to the emergent structures of ecosystems, food webs and communities.

Epigram:

Then see,

At the bottom of all,

The deep peace and simplicity,

As you watch

A single leaf fall.

S.A. Cushman

## 1. Introduction

The world “oecology” was coined by the German zoologist, Ernst Haeckel in the 1860s, in an attempt to define the study of the complex struggle for existence that Darwin had discussed in *On the Origin of Species* [1]. Darwin realized that the tendency described by Malthus [2] for biological reproduction to produce far more offspring that can be supported by the resources in the environment would lead to competition and mortality, and that variation within a species that was more advantageous for survival and reproduction would be selected through the process he named natural selection. Thus, the struggle for existence became seen as the mechanism for evolutionary change and the origin of species. Ecology came to be the term for the study of the interaction between organisms and their environment within the context of Darwinian evolution, and an ecosystem was defined by Tansley [3] as a community of living organisms in conjunction with the nonliving components of their environment, interacting as a system.

Biological evolution fundamentally emerges from the interplay of three things: heritability, structure and metabolism. Different branches of biological science have focused on these, with genetics focusing on the process of heritability and the structures that govern it, molecular and cellular biology, histology and anatomy focusing on structure, and physiology and energetics focusing on metabolism. Biological science has been largely disciplinary and descriptive, with much research elucidating details of structure and function within a narrow scope. One topic that has been very much under studied and largely ignored in evolutionary biology is the overarching context of thermodynamics in controlling all biological processes and the evolution of life.

Most fundamentally, organisms are self-replicating dissipative structures. All structure and non-random aggregation in nature is driven by increasing disaggregation and disorder in the larger system in which it is contained. Simply stated, there are more ways to be disordered than ordered, more ways for something to be broken than fixed, and more ways to be dead than alive. A dissipative structure is something that builds and maintains order by consuming free energy and therefore creating more disorder in its environment. Organisms are dissipative structures, or rather complex systems of dissipative structures. All biochemical reactions are governed in their direction and rate by free energy. A spontaneous reaction occurs when the reaction consumes free energy, transforming highly ordered energy capable of doing work into degraded thermal energy with lower ability to do work.

All the molecules, tissues and systems in an organism’s body are dissipative structures built and maintained by the organism through continued consumption and degradation of order and free energy. Organisms are self-replicating dissipative structures that have evolved through natural selection to maximize their ability to maintain their organization through efficient utilization of energy, which is directly linked to increasing entropy, through the second law of thermodynamics. Living beings have been built by evolution to efficiently consume free energy to maintain homeostasis and engage in reproduction. Organisms are self-replicating dissipative structures.

The definition of an organism is an entity that builds and maintains a structure that has metabolism and heritability. Evolution is the process whereby variation in the structure of organisms have differential fitness in terms of their effectiveness at building and maintaining their structure, efficiently consuming free energy, and effectively reproducing and passing on those heritable variations, leading to change in the frequency of genetic variation and associated change in the characteristics in the population. Biology emerged in the 19th century from the study of natural history, which focused directly on describing the structure and diversity of life. This tradition focused explicitly on the structure and did not directly consider process. Darwin’s theory of evolution was the revolutionary change that refocused biology into the study of evolution and how it drives the emergence of structure and adaptation. However, the true underlying processes of biology are not evolution per se. Evolution by natural selection is a result of the underlying processes of energy flow through self-replicating dissipative structures. I believe that biologists have been over captivated by the structure that emerges from evolution, neglecting to fully understand the processes that drive, control, and governs it. When looking at the ocean, we see waves but do not imagine the existence of water. The central process is dissipation of free energy according to the second law of thermodynamics, and evolution therefore is better conceptualized as the emergence of self-replicating dissipative structures that, through natural selection, become increasingly more efficient at degrading free energy.

This essay is to attempt to reframe the concepts of ecology, ecosystem and evolution in a more generalized context that is consistent with the fundamental physical processes that govern natural change, specifically the flow and degradation of energy and creation of disorder through the second law of thermodynamics. It is the natural continuation of the ideas I first developed in [4], and I believe that there is a profound unity and simplicity in thinking about ecosystems and evolution in this context. The attempt is, in my opinion, to stand the question back on its feet, after seeing it balanced on its head throughout the development of the discipline. The structures of life, to which biologists have focused their attention on describing, measuring, quantifying and explaining, are really the byproduct, the “dissipative structures”, created by the grander universal process of the unwinding of the great spring of order and degradation of useful energy in the universe. I believe that by seeing biology as a thermodynamic process governed by the most fundamental laws of physics brings clarity and an understanding of life, its structure, evolution and meaning, which cannot be obtained from alternative perspectives.

I arrange the essay in several parts. First, I review the development of the second law of thermodynamics and what it means as a central organizing principle of nature. Second, I pivot to the idea of dissipative structures and show that biological organisms are most correctly defined as self-replicating dissipative structures. Finally, I discuss the idea of ecosystems as aggregate networks of interacting organisms—that is, networks of self-replicating dissipative structures. I describe the patterns of ecosystems that consistently emerge as functions of productivity and biomass, such as energy pyramids, food webs and communities, and argue they are most understandable when conceived as emergent properties of the interaction of thermodynamic dissipative structures. It is my hope that this will enable readers from biology, chemistry and physics alike to see the unity between the fields and provide a first step to a unified theory of ecology based on thermodynamics.

## 2. Part 1—Heat, Work, Entropy and Free Energy

Nicholas Carnot is credited with realizing that there was an inherent inefficiency converting heat into work [5]. Joule showed in 1840s that heat was not conserved, but that work could be converted quantitatively into heat. This led to the realization that work and heat are mutually interconvertible, but that heat was not fully convertible into work and that heat is not a substance like water. William Thomson (aka Lord Kelvin) proposed that two laws could be underlying the phenomenon, such that Carnot’s work could be reconciled with Joule. Thus emerged the study and the name of thermodynamics.

Rudolf Clausius also saw the Carnot vs. Joule debate could be resolved if there were two underlying principles of Nature [5]. He speculated on how heat could be explained in terms of the behavior of particles of matter. Ludwig Boltzmann forged the link between the aggregate properties and the behavior of individual particles. Boltzmann perceived that the aggregate interaction among atoms of bulk matter could explain thermal properties and the interchange between work and heat.

There are four laws of thermodynamics. The Zeroth law defines temperature and equilibrium in temperature. The First Law is “energy is conserved”. That energy, rather than heat, is what conserved was the key contribution of Clausius and Kelvin. With the First Law, energy supplanted work as the central principle of physics. Energy was defined as the capacity to do work, and physics as the science of energy. The Second Law recognizes that there is a fundamental dissymmetry in nature: hot objects cool, but cool objects do not spontaneously become hot; a bouncing ball comes to rest, but a stationary ball does not spontaneously begin to bounce. Although the total quantity of energy must be conserved in any process, the distribution of that energy and its utility in doing work changes in an irreversible manner. It is this Second Law of thermodynamics that the ideas of this essay are built around. The third law deals with properties of matter at very low temperatures and states that we cannot bring matter to a temperature of absolute zero in a finite number of steps.

The differences in the effort and sophistication needed to produce heat on the one hand and work on the other is centrally important. Consider first the challenge of producing heat from petrol. All one needs is a match to supply heat sufficient to trigger combustion and oxygen to fuel the spontaneous chemical reactions that release energy from complex organic molecules and produce byproducts of water and carbon dioxide. Consider second the challenge of producing work from petrol. An internal combustion engine is required to propel an automobile, and this engine is a complex machine with many interacting parts built and assembled with high precision and careful engineering. Why is it so much harder to produce work than heat from a given amount of energy?

The fundamental challenge in any biological or technological task is to extract ordered motion from disordered motion, for therein lies the difference between heat and work [5]. To illustrate this idea, the Carnot engine is useful. The Carnot engine is a theoretical device consisting of a working gas confined in a cylinder which can be put in contact with hot source, cold sink and insulated. Only by allowing energy to be discarded into a cold sink does the cycle have any ability to produce work. The price paid to generate work from heat is to discard a portion of that heat. Only if a cold sink is available can the process orchestrate the dissipation of energy to draw off some as work.

The ultimate unification of the concepts of Kelvin, Clausius and Boltzmann are that the simple idea of energy dispersing accounts for all the change that characterizes the world. When we grasp that energy disperses, and only through its dispersal can there be any work, aggregation, organization or structure created, we grasp the fundamental engine of all nature. Work involves coherent motion; heat involves incoherent motion. Only by creating an excess of incoherent motion can we increase coherent motion in any local subsystem.

The Second Law stated in terms of behavior of individual atoms is that energy tends to disperse leading to the dispersal and equilibration of location and energy level among atoms. This is not teleological: all action, order and structure are simply a consequence of the way that particles happen interact and transfer energy among themselves. Cooling is the process through which energy is discarded into the cold sink in a heat engine, and analogous processes of cooling underlie chemical reactions and all other processes that produce or maintain order or concentrate energy [5]. Cooling is the process through which the total energy of the system remains the same, but the distribution of energy spontaneously equilibrates and disperses, and becomes more disordered.

How would one quantify this disorder? The concept of entropy is a critical idea and provides a measure of the disorder of a system. Ludwig Boltzmann, in a simple but elegant equation, expressed entropy in terms of the number of ways particles can be arranged in a system to produce a given macroscopic property. The equation is S = k log W, which states that the entropy of a system is proportional to the logarithm of the number of microstates in a macrostate.

Consider the cooling of a hot object in a cold closed system. Imagine starting this system with activated or hot molecules concentrated in one corner of the system and inactivated cold molecules in the rest. Random jostling of particles results in one activated atom passing some of its energy to a cold inactivated atom. In the context of Boltzmann’s equation, the new value of W is the number of different ways of an atom to be activated by these interactions. The random interactions of molecules produces the spread and dissipation of activation away from the area of initial concentration of thermal molecular motion. There are more ways for the thermal energy to spread out than to stay concentrated in one corner of the system, and therefore the entropy of the system greater than before. As random interactions of the atoms continue, more activated atoms transfer energy to atoms in other parts of the closed system. The maximum entropy occurs when the full system is at thermal equilibrium, with equal density of activated atoms. There is not net flow of energy from one part of the system to another and the system will remain in equilibrium forever, except for chance fluctuations.

This simple idea of random dissipation of order and energy is deceptively profound. Boltzmann’s equation describes the statistical way in which systems evolve. This illustrates the fundamental irreversibility of natural change. All structures and events correspond to the evolution of the universe through successive states of increasing probability, with disordered, dispersed energy states having higher probability than organized and concentrated states.

## 3. Part 2—Organisms Are Self-Replicating Dissipative Structures

All structures and events emerge from the irreversible process of the universe evolving into states of increasing probability, characterized by greater disorder, less concentrated and less usable energy. It has been said that ecosystems are not only more complex than we think, but that they are more complex than we can think (Frank Egler), reflecting the notion that the interaction of vast numbers of organisms in space and time with the structure of their environment is inconceivably complex. The profound idea that emerges from the Second Law is that ecosystems are structured, driven and maintained by the simplest process that can be envisioned. The purposeless random dissipation of energy is the only fundamental process in biology and ecology. All else emerges from it.

Chemical reactions are elaborations of the processes of heat engines [6]. The reason a chemical bond is formed is that the energy of the molecule is less than the energy of the separated atoms. Thus, in chemistry, as in physics, the driving force of natural change is the chaotic, purposeless, undirected dispersal of energy. Both the direction of natural change and the rate at which it is achieved are aspects of the distribution of energy. The direction of natural change depends on the tendency of energy to disperse. The rate depends on the temperature (the total thermal energy in the system) and the abundance and concentration of chemical species. This then produces the Boltzmann probability of activation energy, in which the probability of a reaction occurring which equals e^(-Activation energy/Temperature). So long as reactions are linked such that one change may be constructive and lead to a local reduction in entropy, chemical and biological systems can grow increasingly complex. The key point, however, is that linked to the process that produces order there must be a process that generates more than a compensating amount of entropy.

Josiah Willard Gibbs developed the concept of free energy, which is fundamental to chemistry and to biology. Free energy = (Total energy) − Temperature × (Entropy change). Any decrease in the entropy a system requires that some energy must be released as heat if the reaction is to be spontaneous. Conversely, a reaction that increases entropy can draw in energy as heat and use some to produce work. Although the entropy of the surroundings drops, this is compensated by the increase in entropy in the system.

This is the thermodynamic process that underlies the major biochemical pathways. For example, in photosynthesis photons, which are low entropy packets of energy, strike a membrane in Photosystem II driving the reactions that store energy. In these reactions, most of the energy of the photon is discarded as heat and some is stored in the intermediate product, which is passed to photosystem I, where another photon drives the reaction, again with some energy stored in the chemical bonds of the intermediate product and more energy from the photon discarded as heat. In these reactions, energy is taken from photons to drive chemical reactions up the free energy ladder, storing usable energy, but degrading the quality of the energy of the photon, discarding much of it as heat with much less ability to do work.

It is often asserted that biological organisms violate the second law of thermodynamics, because, unlike nonliving systems, they grow, build tissues and structures, maintain them through homeostasis, and then reproduce themselves, appearing to flout the notion that natural change inevitably increases entropy. However, when the thermodynamic basis of natural change is understood we can see that this is not the case. Photosynthesis is the fundamental driver of life on earth and the source of primary productivity that feeds nearly all organisms. We have seen above that this process of photosynthesis is indeed fully consistent with the Second Law. Energy is degraded in the light reactions, some stored in chemical bonds, but much discarded as heat, increasing the entropy of the universe. In the Calvin Cycle, these chemical bonds are broken, releasing energy, which is in part used to synthesize glucose, but at the cost of a net loss of free energy and increase in the entropy of the universe.

An analogous process of net increase in entropy and decrease in free energy occurs in all other metabolic and biochemical pathways. For example, the citric acid cycle, degrades the energy stored through photosynthesis through a number of intermediate reactions in which oxygen is consumed and carbon dioxide released. The process is essentially “burning” glucose and using some of the energy released by that oxidization to drive creation of high energy bonds in energy currency molecules such as NADH, ATP and GTP. At each step of the process a portion of the energy released in the reaction is degraded and discarded as heat, increasing the entropy of the universe.

Photosynthesis is the primary chemical pathway through which energy enters biological processes, and the citric acid cycle is the primary way it is converted into the small, energy rich molecules that drive the biological processes of life. The main point I wish to make is that in both of these cycles energy is degraded and discarded as heat, and the entropy of the system increased substantially more than the order created by the biochemical processes that are driven by them. Biochemical heat engines drive all biological processes, and these heat engines run by dissipating order and increasing disorder. They function because they transform ordered motion and concentrated energy into disordered motion and dispersed energy.

A dissipative structure is something that builds and maintains order by consuming free energy and therefore creating more disorder in its environment. Dissipative structures arise as a consequence of dispersal and degradation of energy and associated increase in entropy and disorder. The structure is sustained by the flow of energy and as soon as that ceases the structure decays. When the process producing the dissipative structure occurs, the rate of generation of entropy in the universe is increased because energy is being dissipated more rapidly by the dissipative structure than it would in its absence. In the case of organisms, the entropy of the universe is increasing more rapidly as a result of photosynthesis and the biochemical pathways of metabolism and tissue growth than it would if the photons had fallen on inanimate earth. As soon as the source of energy is removed (photons from the sun), the dissipative structures of organisms will die and decompose. They are thermodynamic instabilities driven by the flow of energy and the transduction and degradation of that energy.

Organisms are dissipative structures, or rather complex systems of biochemical dissipative structures. All biochemical reactions are governed in terms of their direction and rate by free energy. All the molecules, tissues and systems in an organism’s body are dissipative structures built and maintained by continued consumption and degradation of order and free energy. Organisms are machines built by evolution to efficiently consume free energy to maintain homeostasis and engage in reproduction. Organisms are self-replicating dissipative structures, and evolution is the process through which variants of these self-replicating dissipative structures that are better able to efficiently consume free energy and create more disorder and entropy in the system. This is where the common perceptions of biology and nature must be stood back up on their feet, after lying so long on their heads. Life does not dissipate energy; the dissipation of energy causes the structure of life to emerge. Life is an emergent property of a system that has evolved to dissipate energy efficiently and increase entropy, not the other way around.

## 4. Part 3—Ecosystems Are Networks of Self-Replicating Dissipative Structures

Ecosystems are linked series of dissipative structures with heat engine dynamics driven by random dissipation of energy and increasing entropy. The structure and composition of ecosystems across scales are emergent dissipative structures driven by the flow of energy and the increase in entropy. Analogous to the transformation and degradation of energy and increase in entropy within organisms discussed above, there is a similar pattern of decrease in useable energy through the food web and energy pyramid. Secondary productivity is the rate of production of new biomass by heterotrophic organisms. Heterotrophs obtain matter and energy by consuming plant material or indirectly from plans by eating other heterotrophs. In ecosystems, as energy is transferred among trophic levels energy becomes degraded, dispersed and diminished from higher quality to lesser quantity. As a general rule of thumb, there is a 90% loss of usable energy between trophic levels in terrestrial foodwebs, and somewhat less loss between trophic levels in aquatic food webs. This results in a pyramidal structure in which the productivity of plants is a broad base, upon which a much smaller productivity of primary consumers depends, upon which again increasingly attenuated production of secondary and tertiary consumers depend [7]. This very steep loss of usable energy through the food web is why in terrestrial systems it is rare to find very many cases of more than three or four trophic levels [8]. In aquatic systems, due to the higher efficiency of trophic transfer, and stronger niche partitioning among levels of consumers, it is more common to have additional trophic levels.

The energetic loss between trophic levels is a direct analogy to the concept of thermodynamic cooling that drives the heat engine model of extracting work from heat. Specifically, herbivores do not eat all the plant biomass produced. Second, not all the plant biomass that is eaten by herbivores is assimilated and available for incorporation into consumer biomass. Much of it is lost as feces, urine and respiration products. Third, not all of the energy assimilated is converted to biomass. There is inherent thermodynamic inefficiency to metabolism, and subsequent linked biochemical pathways using metabolic products also have thermodynamic inefficiencies, such that a large portion of the assimilated energy is used for homeostasis and does not build new biomass.

The proportions of net primary productivity that flow along each of the possible energy pathways in an ecosystem depend on transfer efficiencies that describe how much energy at one step in the pathway is transferred to the next step. Consumption efficiency is defined as the percentage of total productivity available at one trophic level that is consumed by the trophic compartment at the next higher level up the trophic ladder. The pattern of consumption efficiency varies among ecosystems, with approximately 5% in forests, 25% in grasslands and 50% in plankton-dominated systems [9,10]. These differences in consumption efficiency are related to the degree to which the primary productivity can be utilized. Forest vegetation is often out of reach and woody, reducing utilization whereas, plankton are generally available in the water column, which increases utilization.

Assimilation efficiency is the percentage of the food energy taken into the guts of consumers at a trophic level which is assimilated across the gut wall and becomes available for growth, homeostasis, or movement. Assimilation efficiencies are generally low for herbivores (20–30%) and high for carnivores (~80%) due to the inherent difficulty of assimilating plant material in comparison with assimilating animal material [11]. Seeds and fruit can be assimilated with an efficiency of as high as 60–70%, leaves 50%, whereas assimilation of woody matter can be as low as 10%.

Production efficiency is the percentage of assimilated energy which is incorporated into new biomass. Production efficiency varies among taxonomic groups. Invertebrates generally have relatively high production efficiencies (30–40%), with relatively little energy lost in respiration. Ectotherm vertebrates, which do not regulate their body temperatures metabolically, have intermediate production efficiencies (~10%), whereas ectotherms, which expend a large proportion of energy maintaining thermal equilibrium, generally have a production efficiency of only 1–2%.

The paragraphs above describe the energetics of ecosystems as aggregate systems. However, it is important to appreciate that ecosystems have spatial structure and temporal dynamics that drive pattern-process relationships that govern distribution, abundance, fitness and evolution of organisms. Put shortly, ecosystems are the stage on which the play of evolution is acted (Robert May), and the spatial and temporal variation of that stage fundamentally affects ecological and evolutionary processes [12]. As a result of this realization, the topic of ecological thermodynamics has recently arisen in the field of landscape ecology [4,12,13,14,15,16,17,18,19,20,21,22,23,24]. Most of this work has focused on developing measures of configurational spatial entropy to quantify landscape patterns [12,13,14,15,16,17,18,19,20,21,22,23,24], and initial attempts to link these to thermodynamic processes [4,15]. This has provoked debate, with [25] asserting that thermodynamics has nothing to do with landscape ecology and the entire endeavor should be abandoned, and [26] countering that “From atoms to the cosmos, entropy is everywhere. Certainly, ecosystems, landscapes and their pattern-process relationships are not uniquely exempted from the laws of thermodynamics. Ispo facto, landscapes are thermodynamic systems. As thermodynamic systems, landscapes therefore have entropy”.

This debate is a motivation for this essay. Applications of entropy in landscape ecology have been “top down”, in terms of applying entropy measures to the patterns of landscapes, which are several orders of magnitude and many organizational levels away from the scales at which classical thermodynamics and entropy studies traditionally take place (e.g., molecules and heat). The main purpose of this essay is to link entropy and the second law to all biological process and structure, from molecules to ecosystems and landscapes. In [4], I suggested that “The application of thermodynamic entropy concepts in landscape ecology has not addressed the true thermodynamic nature of the actions of dissipative structures across scales, and this has been limited by failure to measure energy transformations, changes in free energy, changes in configurational entropy of landscape mosaics. As a result, there has been a nebulous and inconsistent application and interpretation of these ideas in the field.” I believe that the identification of thermodynamics and the entropy concept as the core process that drives natural change across scales is the key to unifying and clarifying physical and biological science.

## 5. Conclusions

In the thermodynamic perspective advocated here, ecosystems are perhaps best considered to be networks of self-replicating dissipative structures (organisms) that have emergent properties of dissipative structures themselves. The predictable pattern of trophic levels, food webs, utilization and assimilation and production efficiencies across ecological systems indicate the tight control that energetics has on the structure of communities and ecosystems. A community or an ecosystem therefore is a kind of dissipative structure, in which networks of other dissipative structures (organisms) evolve to maximize their fitness in survival and reproduction, which in thermodynamic terms means the efficiency in which they can utilize, assimilate and use energy, and which, when stood back upright thermodynamically, means that ecosystems are the emergent effect of the cascade of energy through the biosphere. Communities and ecosystems are emergent properties of a system that has evolved to most efficiently dissipate energy and increase entropy. By focusing on the fundamental entity (energy), and the fundamental process (dissipation and disordering of energy and increasing of entropy) we are able to have a much clearer and powerful understanding of what life is, from the level of biochemistry, to evolution, to the nature of the organism itself, to the emergent structures of ecosystems, food webs, communities and landscapes.

Instead of standing it on its head, and focusing on the transient, idiosyncratic and variable dissipative structures, it is more unifying and clarifying to understand the profound simplicity underlying ecological systems. It is the simple process of maximizing the local efficiency of increasing entropy through systems of dissipative structures. The only process is the random degradation of order and decrease in usable energy in every and all actions. So, contrary to the statement that “ecosystems are not only more complex than we think, they are more complex than we can think,” it might be more illuminating to understand that they are in fact not only governed by the simplest process we imagine, but they are governed by the simplest process we can imagine. It is the central process that governs nature, the random dissipation of useful energy and the degradation of order as the entropy of the universe increases with the unwinding of the cosmic clock.

This may appear nihilistic from the dominant humanistic epistemology of our society. At the heart of this vision appears to be emptiness, the realization that all objects, identities and concepts of self are transient and illusory. It may seem to imply that we do not live life, but that life lives us, and that life itself is merely physio-chemical structures emerging from the dissipation of energy and relentless increase in entropy.

To respond to these potential criticisms, let me return to Boltzmann and his own words which show his grand vision of the unifying simplicity of entropy and the second law, and which, I believe, properly understood, turns the apparent nihilism on its head. “The general struggle for existence of animate beings is not a struggle for raw materials, these for organisms are air water & soil, all abundantly available, nor for energy which exists in plenty in the sun and any hot body in the form of heat, but rather a struggle for entropy [exergy], which becomes available through the transition of energy from the hot sun to the cold earth”. In this quote, we see Boltzmann framing life fundamentally as a thermodynamic system driven by entropy. Boltzmann also wrote: “Thermodynamics, correctly interpreted, does not just allow Darwinian evolution; it favors it”. and “Available energy is the main object at stake in the struggle for existence and the evolution of the world”. He stated that “In my view all salvation for philosophy may be expected to come from Darwin’s theory”. Boltzmann unequivocally writes “All structures and events correspond to the evolution of the Universe through successive states of increasing probability”. He emphatically asserts “Bring forward what is true, Write it so that it is clear, Defend it to your last breath!” Boltzmann advanced and defended the grand implications of his work on entropy indeed to his last breath, and beyond. By having it inscribed on his tombstone, he made s = k log w his final and abiding testament to the world.

This paper may appear overly philosophical and without concrete application; however, the purpose is to suggest a new perspective on biology and evolution, with energy, entropy and the emergence of dissipative structures as the focus. Future work should add concrete development to these ideas by exploring and simulating dynamics of biological evolution, organism structure and ecosystem processes in a thermodynamic framework.

I believe that a unifying understanding of our world comes from the integration of Darwin’s and Boltzmann’s theories. The unification of entropy, ecology and evolution seems to present a grand opportunity to clarify the natural sciences and philosophy. I close with a rephrasing of Darwin’s famous last sentence from *On The Origin of Species* in terms of entropy and the second law. There is a grandeur to this view of things, with its simple, undirected process underlying all creation and all structure, and whilst the cosmos has gone on unwinding, according to the second law of thermodynamics, endless forms are being and will be emergent.

## Data Availability

No data are presented in this paper.
